# p63 at the Crossroads between Stemness and Metastasis in Breast Cancer

**DOI:** 10.3390/ijms20112683

**Published:** 2019-05-31

**Authors:** Veronica Gatti, Lucilla Bongiorno-Borbone, Claudia Fierro, Margherita Annicchiarico-Petruzzelli, Gerry Melino, Angelo Peschiaroli

**Affiliations:** 1 Department of Experimental Medicine, TOR, University of Rome Tor Vergata, 00133 Rome, Italy; veronica_gatti@yahoo.it (V.G.); lucilla.bongiorno@uniroma2.it (L.B.-B.); claudia_fierro@libero.it (C.F.); melino@uniroma2.it (G.M.); 2 Istituto Dermopatico dell’Immacolata, IDI-IRCCS, 00163 Rome, Italy; m.annicchiaricopetruzzelli@idi.it; 3 Medical Research Council, Toxicology Unit, University of Cambridge, Cambridge CB2 1PZ, UK; 4 National Research Council of Italy, Institute of Translational Pharmacology, 00133 Rome, Italy

**Keywords:** p53 family, epithelial tumors, TNBC, metastasis, cancer stem cell

## Abstract

After lung cancer, breast cancer (BC) is the most frequent cause of cancer death among women, worldwide. Although advances in screening approaches and targeted therapeutic agents have decreased BC incidence and mortality, over the past five years, triple-negative breast cancer (TNBC) remains the breast cancer subtype that displays the worst prognosis, mainly due to the lack of clinically actionable targets. Genetic and molecular profiling has unveiled the high intrinsic heterogeneity of TNBC, with the basal-like molecular subtypes representing the most diffuse TNBC subtypes, characterized by the expression of basal epithelial markers, such as the transcription factor p63. In this review, we will provide a broad picture on the physiological role of p63, in maintaining the basal epithelial identity, as well as its involvement in breast cancer progression, emphasizing its relevance in tumor cell invasion and stemness.

## 1. Introduction

Although promising targeted therapeutic agents have been developed over the past two decades, breast cancers (BCs) remain the most frequent cause of cancer death among women worldwide, after lung cancer [1,2]. Based on the histological and molecular features, BCs are molecularly defined into four different subgroups—Luminal A and B, HER2 positive, and triple-negative breast cancers (TNBC). Luminal BCs represent the vast majority (60–80%) of BC cases in developing countries, and they are further classified as Luminal A and Luminal B [3,4,5].

Luminal A are characterized by the expression of estrogen receptors (ER) and progesterone receptors (PR) and generally have the best prognosis among different BC types, responding well to the ER antagonist tamoxifen and aromatase inhibitors [6,7]. Luminal B tumors are ER-positive but PR-negative, and can be either positive or negative for the expression of human epidermal growth factor receptor 2 (HER2/ERBB2). In contrast to Luminal A, Luminal B tumors are more aggressive and do not respond well to endocrine treatment [8].

HER2-positive tumors display an overexpression or amplification of the HER2 oncogene and can be clinically treated with the anti-HER2 monoclonal antibody trastuzumab, in combination with conventional chemotherapy, improving the survival rate by 30% [9,10,11,12].

TNBC are generally defined on the basis of the lack of expression of hormone and the HER2 receptors [13]. The absence of these clinically actionable targets implies that TNBC therapy relies merely on standard approaches, such as radiotherapy, and a combination of chemotherapeutic agents like anthracyclines, alkylating agents, taxanes, or platinum salts [13]. In addition, genetic and molecular profiling has unveiled the high heterogeneity of TNBC, a feature that likely limits the clinical management of these tumors and, as a consequence, TNBC prognosis [5,14,15,16]. TNBC is indeed the breast cancer subtype displaying the worst prognosis, and TNBC patients display low rates of disease-free survival, overall survival, and five-year survival.

**Figure 1:**
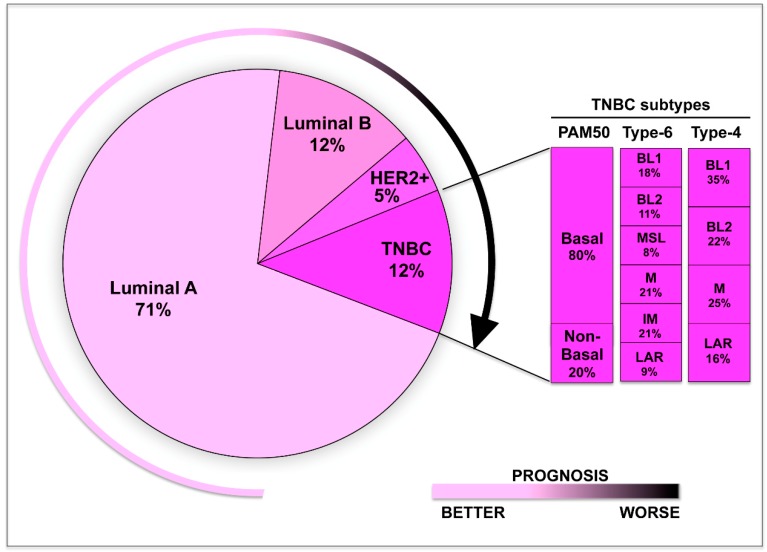
Schematic model showing the molecular heterogeneity of triple-negative breast cancer (TNBC). Based on the expression profile, TNBC can be classified into distinct subtypes— Basal-like 1 (BL1), basal-like 2 (BL2), immune modulatory (IM), mesenchymal (M), mesenchymal stem–like (MSL), and luminal androgen receptor (LAR). The percentage of each subtypes is also indicated.

Over the past years, genomic, transcriptomic, proteomic, and epigenomic profiling have allowed to classify TNBC into distinct clinical and molecular subtypes [15]. Lehmann and colleagues have subdivided TNBC into six subtypes—basal-like 1 (BL1), basal-like 2 (BL2), immune modulatory (IM), mesenchymal (M), mesenchymal stem–like (MSL), and luminal androgen receptor (LAR) [16] (see Figure 1). Subsequent studies of the same group have refined this classification on the basis of the contribution of the stromal and immune cells, to the gene expression profiles of the IM and MSL subtypes, respectively [17]. Therefore, TNBC might be simply categorized into four subtypes—BL1, BL2, mesenchymal, and LAR (Figure 1).

Each TNBC subtype displays distinct clinical features, response to neoadjuvant chemotherapy, and relapse-free survival rates, likely reflecting specific genetic and molecular features associated with each subgroup. Basal-like BCs are characterized by the expression of basal epithelial markers, including the transcription factor p63. The *TP63* gene belongs to the p53 family of transcription factors, which includes p63 itself, p73, and the canonical tumor suppressor gene p53 [18]. The activity of members of this family is intimately linked with the pathogenesis of different epithelial tumors, including BC. p53 is considered the guardian of the genome, since it preserves the genome integrity by inducing cell cycle arrest, apoptosis DNA repair, or metabolic adaptation, in response to different stresses [19,20,21,22]. In TNBC, *TP53* mutation is frequently observed with a mutational rate of 80% of cases, and is associated with a more aggressive phenotype [23,24]. Besides losing their tumor suppressor functions, some p53 mutations, the so-called Gain-Of-Function (GOF) mutations, acquire oncogenic functions promoting metastatic dissemination and drug resistance [25,26,27,28,29]. Although the *TP63* gene is rarely mutated in TNBC, several studies have clearly demonstrated the relevance of p63 activity in controlling several tumor-associated events, especially in basal-like subgroups. In the next paragraphs, we will portray a general view of the physiological role of p63, in regulating mammary gland homeostasis, as well as the p63-activated pathways involved in breast cancer progression.

## 2. *Tp63* Gene Architecture

**Figure 2:**
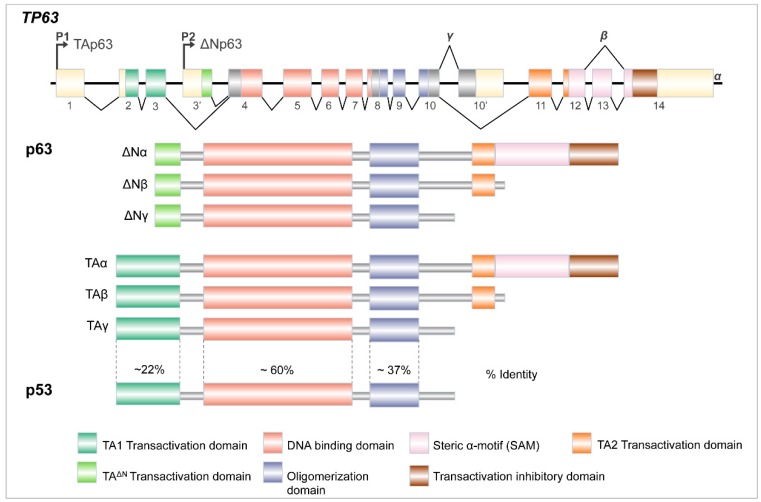
The *TP63* gene architecture. *TP63* gene encodes several proteins, thanks to two distinct promoters (P1 and P2) and differential splicing events at the 3′ end of its RNA (see text for details).

p63 is the most ancient member of the p53 family of transcription factors, which also includes p73 [18,30]. Human *TP63* gene is located on chromosome 3q27–29, which consists of 15 exons spread over 220 kilobases, and can generate multiple isoforms, thanks to different promoter usage and alternative splicing at the C-terminus (Figure 2). p63 protein functions as tetramer and each monomer is arranged in structurally and functionally defined protein domains, closely related to p63’s well-known homolog p53. All p63 isoforms encode a core DNA-binding domain (DBD), which is about 60% identical at the DBD of p53 and, as a consequence, can bind to the canonical p53 response element, as well as p63-specific DNA-binding sites. Next to the DBD and common to all p63 isoforms, there is an oligomerization domain (OD) that shares approximately 37% of identity with p53. This allows homo- and hetero-oligomerization between different isoforms of p63 itself and other p53-family members [30,31,32].

p63 can be transcribed, starting from two distinct promoters, one (P1) arranged immediately before the first exon and the other (P2) located in the third intron. The choice between P1 and P2 yields two different proteins that might include (TA) or not (ΔN), the N-terminal p53-homologous TransActivation domain (TA1). In addition, the 3′ end of both TA and ΔN transcripts can undergo alternative splicing events, generating three different C-terminal isoforms (α, β, and γ). The full-length α isoforms contain two distinctive domains: a putative protein–protein interaction domain found in other transcription factors and signaling proteins called Sterile alpha-motif (SAM) and a transcription inhibition domain (TI), Importantly, the TI domain promotes a closed conformation, through the binding with TA1, restraining the transcriptional activity of the TAp63α isoform in the physiological condition [33,34]. Conversely, upon genotoxic stress, specific post-translational modifications release the T1–TA binding, activating TAp63α transcriptional activity. The physiological relevance of this regulation has been clearly demonstrated in the oocytes, where DNA damage induces TAp63α transcriptional activity toward pro-apoptotic genes, thus preserving, the female germline [35,36].

Due to the absence of a canonical TA1 domain, the ΔNp63 isoforms have been initially described as dominant negative factors counteracting the TAp63 and p53 functions [32]. However, subsequent studies have clearly demonstrated that, in addition to this function, ΔNp63 can act as a sequence-specific transcriptional activator or repressor. In detail, the ΔNp63α isoform can transactivate specific target genes, due to a second transcription activation domain (TA2), arranged within exons 11 and 12, and a residual transactivation activity in the N-terminal truncated region (TA^ΔN^) [37,38,39,40]. In addition, ΔNp63 acts as a potent transcriptional repressor interacting with diverse epigenetic factors, such as histone deacetylases HDAC1 and HDAC2, the chromatin remodeling protein complex SRCAP, the SWI/SNF subunit ACTL6A, and the histone lysine methyl transferase SETDB1, which have all been implicated in the ΔNp63-mediated transcriptional repression [41,42,43,44].

Multiple studies have analyzed the role of p63 isoforms in regulating physiological and pathological processes, such as tumor development. The general view emerging from these studies is that TAp63 acts as a tumor suppressor, while ΔN isoforms promote cancer cell survival and proliferation. In the next paragraphs, we will describe the physiological function of p63 isoforms in maintaining mammary gland homeostasis, and deciphere the p63-related pathways involved in breast tumor initiation and progression. If not specified, we used TAp63 and ΔNp63 to indicate the α isoforms, which are the most expressed p63 isoforms in epithelial tissues. 

## 3. Physiological Role of p63 during Mammary Gland Development

**Figure 3:**
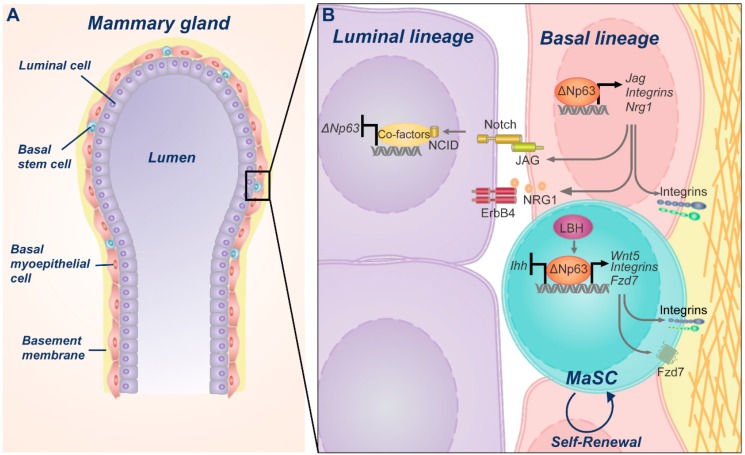
Schematic model of ΔNp63 physiological role in mammary gland. (**A**) Representation of mature mammary gland duct showing an inner layer of luminal cells surrounded by a network of basal myoepithelial cells and scattered stem cells. (**B**) Magnification of the inter- and intra-cellular signaling pathways orchestrated by ΔNp63, aimed at preserving the stem cell self-renewal potential, as well as determining the luminal or basal fate.

Mammary gland consists of a bilayered epithelium, made of two distinct cell compartments, luminal and basal. The luminal compartment consists of a single layer of apically oriented, polarized epithelium surrounding the lumen of the gland ducts, which includes structural ductal cells and milk-producing alveolar cells. The basal compartment includes rare stem cells and myoepithelial cells that are arranged in a network around luminal cells and have a contractile function in milk ejection (see Figure 3A) [45,46]. Mammary gland is a plastic organ that undergoes several important changes during the lifetime. It reaches full development during puberty, goes through massive side branching of the ducts during pregnancy and undergoes involution, upon breastfeeding suspension. The impressive regenerative potential of the mammary gland epithelium implies the existence of a mammary epithelial cell population endowed with self-renewing properties. Many studies have indeed proven that mammary stem cells permanently residing within the basal compartment of the mammary gland can generate lineage-committed progenitors (luminal and myoepithelial progenitors), which in turn differentiate into myoepithelial, ductal, or alveolar cells [47,48,49].

Several lines of evidence have clearly indicated that p63 activity is critically involved in sustaining the proliferative potential and self-renewing capacity of mammary epithelial stem cells (see Figure 3B) [50,51,52,53]. The initial observation impinging on a fundamental role of p63, in mammary epithelial homeostasis, arises from the analysis of mice that are genetically devoid of all p63 isoforms. *TP63* null mice display a lack of all squamous epithelia and their derivatives, including mammary glands [52,54]. These defects have been ascribed, at least in part, to a loss of the proliferative potential of the epithelial stem cells and suggest a role for p63 in stemness maintenance [50,52,53]. Remarkably, the genetic selective deletion of ΔNp63 isoforms recapitulates the mammary gland defects observed in the p63 global knock-out mice, strongly indicating that ΔNp63 is the major p63 isoform governing mammary glands morphogenesis [50,55]. This notion has also been confirmed by additional data, showing that ΔNp63 is the major p63 isoform expressed in the epithelial stem cells, where it drives organ reconstitution and ductal branching potential, after mammary epithelial cells transplantation into a cleared fat pad [50].

A relatively recent study suggests that TAp63 might also participate in controlling mammary stem cell activity. Indeed, it has been shown that mammary glands deficient for TAp63 have an increased regenerative potential and display an enrichment of mammary epithelial cells harboring stem-like properties [56]. These data suggest that TAp63 activity might restrain the self-renewing capacity of mammary stem cells, and imply that the p63 isoforms expression (TA and ΔN) needs to be finely regulated in distinct cell compartments. Accordingly, it has been shown that hedgehog (Hh) signaling controls the beginning and the progression of the mammary regenerative cycle by differentially inducing the expression of ΔNp63 and TAp63 isoforms in stem and progenitor cells, respectively [57]. The segregation of p63 isoforms is responsible for the TAp63-dependent induction of the Indian hedgehog (Ihh) in progenitor cells and Ihh repression by ΔNp63 in stem cells. This circuit ensures a tightly regulated balance between a quiescent state maintenance and progenitors maturation.

Although these data suggest that TAp63 isoforms might also contribute to regulate the self-renewing capacity of mammary cells, diverse studies have clearly highlighted the relevance of ΔNp63, in controlling molecular pathways involved in the regenerative potential of mammary epithelium, such as the WNT-β/catenin pathway (see Figure 3B). ΔNp63 activity intersects WNT signaling at multiple levels. ΔNp63 and WNT pathway cooperates in the promotion of stemness, through a direct regulation of the FZD7 receptor and WNT5B ligand expression by ΔNp63 [50]. The WNT/β-catenin target gene *LBH* (Limb, Bud and Hearth) is able to drive the expression of ΔNp63 and to repress that of TAp63. LBH’s capability for maintaining a basal mammary stem cell state and to repress luminal differentiation, requires ΔNp63 expression, suggesting that ΔNp63 activity is pivotal to control cell fate determination and maintain the basal cell phenotype [58]. Accordingly, ΔNp63 knockdown in primary human breast epithelial cells, with basal characteristics, induces a decrease of the basal markers, such as cytokeratin 14 (CK14) and integrin-α6 (ITGA6) together with the appearance of luminal markers [51]. Reciprocally, ectopic expression of ΔNp63 in isolated human luminal cells or in adult murine luminal cells, in vivo, is sufficient to induce a transition towards a basal cell fate [51,59]. The importance of ΔNp63 in sustaining epithelial basal identity is also supported by the finding that the Bromodomain-containing protein 4 (BRD4) maintains a basal epithelial phenotype by regulating the expression of epithelial-specific genes, including *TP63* [60].

Furthermore, *BRCA1*, a DNA repair gene involved in mammary gland differentiation and implicated in the pathogenesis of basal-like breast cancer [61,62], is able to drive the expression of ΔNp63 in basal cells and sustain NOTCH signaling in luminal cells [63]. Importantly, NOTCH signaling is fundamental to commit progenitor epithelial cells towards a luminal phenotype [51]. Mouse mammary epithelial cells bearing inactive NOTCH signaling, showed an impaired luminal lineage differentiation. Notably, NOTCH activity in determining luminal fate specification requires the NOTCH-dependent inhibition of ΔNp63 expression. ΔNp63 expression inhibits luminal cell differentiation and prevents overall reconstruction in mammary gland transplantation experiments. NOTCH-ΔNp63 crosstalk is further complicated by the evidence that ΔNp63 promotes mammary stem cell quiescence, by increasing the expression and activity of NOTCH 3 [64], suggesting that specific regulation of different NOCTH receptors might regulate the fine balance between quiescence and commitment. Collectively, these data indicate that BRCA1, ΔNp63, and NOTCH signaling takes part in a highly regulated crosstalk, ensuring the proper balance between stemness, cell fate specification, and differentiation.

ΔNp63 activity as regulator of stemness and progenitor commitment is not restricted to basal-specific roles, but also modulates the homeostasis of specialized mammary cells, during lactation and post-lactational involution. Pregnancy-specific selective deletion of *TP63* in the basal cells is associated with a complete lactation failure, due to the impaired proliferation and differentiation of luminal cells. At the molecular level, ΔNp63 induces the expression of the EGF family member Neuregulin 1 (NRG1), which triggers a paracrine signaling involving luminal ERBB4/STAT5A activation, leading to luminal progenitor proliferation, alveolar differentiation, and milk production [65].

During post-lactational involution, ΔNp63 mediates the survival of Parity-Induced Mammary Epithelial Cells (PI-MECs), a heterogenous population that escapes apoptotic cell death during post-lactational involution survival. In detail, ΔNp63 represses the expression of the STAT3-positive regulator oncostatin M and promotes the expression of NRG1 and NRG2 that, activating the pro-survival homolog STAT5, opposes the STAT3-induced apoptotic pathway [66,67].

In conclusion, these data clearly indicate a remarkable role of the ΔNp63 in controlling molecular circuits involved in epithelial stemness and cell fate specification, ensuring the regenerative potential and plasticity of the mammary gland epithelium. In the next paragraphs, we will describe how p63 isoforms exploit several interrelated molecular pathways, to finely regulate breast tumor progression.

## 4. ΔNp63 Oncogenic Pathways in Breast Cancer

**Figure 4:**
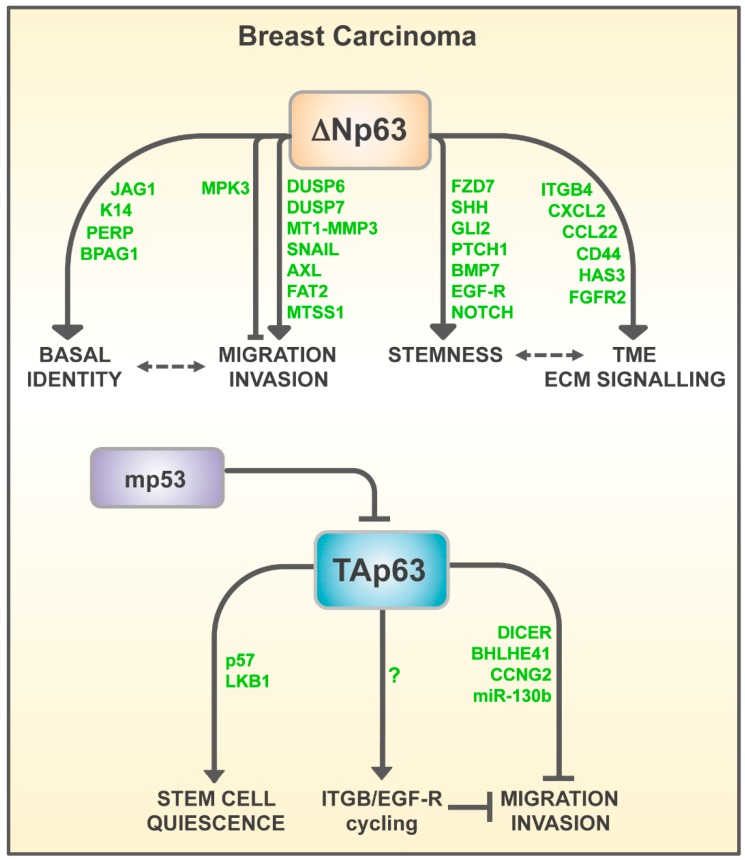
Schematic model of p63-dependent routes involved in breast tumor progression (see text for details).

In the past years, various transcriptional effectors of ΔNp63-driven tumorigenesis in epithelial tumors have been identified and characterized. These ΔNp63 transcriptional targets have been deeply investigated in squamous cell carcinoma, where ΔNp63 acts as an oncogene by regulating expression of diverse tumor-related proteins, critically involved in the extracellular matrix (ECM) remodeling, growth factors-mediated signaling, and tumor microenvironment (TME) remodeling [68]. It is reasonable that these oncogenic routes might also be involved in the pathogenesis of breast carcinoma, contributing to enhancement of proliferation, stemness, and survival of breast tumors. In the following sections, we will focus our attention only on those ΔNp63 transcriptional targets, which have been formally proved to play a critical role in breast carcinogenesis, emphasizing their role in controlling tumor metastasis and cancer cell stemness (see Figure 4).

### 4.1. ΔNp63 in Metastatic Dissemination

Metastasis represents the end-product of a multistep cell-biological process in which cancer cells acquire the ability to invade organs that are anatomically distant from the primary site. The metastasis cascade is modulated by many factors and epithelial-mesenchymal transition (EMT) has been identified as an important process for enhancing the invasive and migratory capabilities of tumor cells [69,70]. By maintaining epithelia identity, ΔNp63 should exert an anti-metastatic action that is able to repress the mesenchymal traits, thus inhibiting the EMT process. Accordingly, in squamous cell carcinoma, which predominantly expresses the ΔNp63 isoforms, p63 depletion led to the upregulation of mesenchymal markers associated with an increase of tumor invasion and metastasis [71]. However, a mesenchymal phenotype can be incompatible with growth in distant tissues [72,73] and basal-like breast cancers, which are intrinsically motile, can collectively invade the surrounding stroma, while maintaining epithelial features [74]. Furthermore, in spontaneous breast-to-lung metastasis models, the EMT process is not required for tumor cell dissemination but rather is implicated in modulating tumor chemoresistance [75,76]. Therefore, it is not surprising that conflicting evidence on the role ΔNp63 in regulating cell motility and invasiveness, has been reported. Here, we will describe some evidence arguing for the pro- or anti-metastatic action of ΔNp63 in breast tumors.

The idea that ΔNp63 acts as a pro-migratory factor in breast cancer cells has been substantiated by two relatively recent papers which showed that ΔNp63 is targeted by different oncogenic signaling, in order to enhance cell migration and invasion [77]. Yoh and colleagues reported that the expression of H-RasV12 oncogene in two non-transformed mammary epithelial cell lines (MCF10A and MCF12A) downmodulates the ΔNp63 expression. This effect is associated with the induction of EMT, increased cell motility, and increased invasive behavior. Importantly, silencing of ΔNp63, recapitulates the H-RasV12-mediated pro-migratory action, arguing that ΔNp63 is a critical effector of the oncogenic activity of H-Ras. Although through a different mechanism, a further study demonstrated that activation of PI3Kinase or HER2 overexpression—two genetic events commonly observed in breast cancer [78,79]—positively regulate cell motility and tumor metastasis by targeting ΔNp63 [80]. At the molecular level, HER2 and PI3Kinase activates AKT, which in turn phosphorylates and inhibits nuclear translocation, as well as the transactivation activity of FOXO3a, a positive regulator of ΔNp63 transcription.

In contrast to these evidence, Vasilaki and colleagues reported that in p53 mutant breast cancer activation of Ras or TFGβ signaling induces the degradation of mutant p53, releasing ΔNp63 transcriptional activity towards two target genes, dual specificity phosphatase 6 and 7 (DUSP6 and DUSP7) [81]. This Ras-ΔNp63-DUSPs circuit, promotes EGF-R-induced or TFGβ-induced breast carcinoma migration and invasion. Although this pathway unveils a potential molecular link between oncogenic events and an increased ΔNp63 activity, the proposed molecular mechanism is barely reconcilable with the oncogenic function exerted by GOF p53 mutations in TNBC. Furthermore, it has been reported that in MDA-MB-231, a highly invasive TNBC cell line expressing mutant p53, the ectopic expression of ΔNp63 is able to negatively modulate invasive behavior through the transcriptional regulation of MPK3, a regulator of ERK1/2 activity [82].

In addition, in prostate and breast cancer cell lines, ΔNp63 restrains the migratory and invasive abilities of cancer cells, by driving the expression of miR-205, an important regulator of EMT [83,84,85]. On the other hand, miR-205 is also able to target p63 mRNA, creating a regulatory feedback loop, which contributes to the regulation of anti-Her2/EGFR therapy response [86]. These findings suggest that in some cellular context, and in specific genetic background, ΔNp63 might restrain cell motility and invasion of breast cancer cells.

In contrast with these data, numerous reports unveiled a pro-migratory and pro-invasive action of ΔNp63 in breast tumors, and different ΔNp63 transcriptional effectors underlying these effects have been identified.

Lodillinsky and colleagues identified the membrane-type 1 membrane-anchored matrix metalloproteinase (MT1-MMP), an important protease involved in tumor invasion [87,88], as a ΔNp63 target gene regulating the invasive properties of basal-like breast cancer cells [89]. During the transition between in situ to invasive carcinoma, MT1-MMP-ΔNp63 circuit is specifically activated in invasive cell clusters. In agreement with these data, ΔNp63 expression has been also found at the edge of microinvasive and invasive tumor breast xenografts, suggesting that ΔNp63 activity might be locally upregulated in invading cells. In addition to MT1-MMP, ΔNp63 directly regulates the expression of another metallopeptidase, MMP13, although the relevance of this pathway in breast tumor has not been demonstrated [90].

Pearson’s lab also confirmed a pro-metastatic role of ΔNp63 in breast cancer [91,92]. In MCFDCIS and HCC1806 cells, ΔNp63 silencing markedly reduces tumor cell motility by transcriptionally regulating the expression of the transcription factor Snail, the EMT-related tyrosine kinase Axl, and the transmembrane protein FAT2. The relevance of these ΔNp63-mediated transcriptional pathways in breast tumors have been confirmed by the worse prognosis of basal-like breast cancer patients displaying high expression of p63 and Slug, or p63 and FAT2. Accordingly, in basal-like breast tumors, high ΔNp63α expression in a p53-mutated genetic background is associated with a shorter overall survival [81].

Another ΔNp63 target gene potentially involved in breast tumor progression is the Metastasis Supressor 1 (*MTSS1*), also known as Missing in Metastasis, or *MIM* or *BEG4*. *MTSS1* is a gene involved in the regulation of actin-based cytoskeleton organization, whose tumor-related function is controversial, since it might act as pro-or anti-metastatic factor, depending on the tumor context [93,94,95]. In some breast cancer cell lines, ΔNp63 directly drives the expression of MTSS1, enhancing the cellular migration and cytoskeleton rearrangements [81]. Notably, in three human breast tumors datasets, the MTSS1/p63 co-expression is a negative prognostic factor on patient survival [96]. Although these data are far from being conclusive, they potentially suggest a molecular effector of the pro-migratory function of ΔNp63, at least in some human breast tumors.

As mentioned above, ΔNp63 expression is restricted to the tumor periphery, in coincidence with the invading cell cluster. In agreement with these data, an elegant study of Ewald’s lab has identified p63 as a critical basal epithelial gene controlling the collective invasion process of several breast cancer subtypes [74]. Collective invasion is a process in which tumor cells invade the ECM, cohesively, as a multicellular unit. By exploiting mouse models of the luminal-type and basal-type breast carcinogenesis, Cheung and colleagues found that cells leading collective invasion, display a conserved, basal epithelial gene expression program that is necessary to exploit the invasive process. In detail, invading tumor cells activate the expression of CK14 and p63 (likely the ΔNp63 isoform), which are required for breast cancer cells to invade the surrounding tissues [74]. It is likely that during the collective invasion of p63 transcriptional activity, it is necessary to sustain a basal epithelial program, in order to maintain a hybrid epithelial/mesenchymal state, which is more permissive to invasion, with respect to a fully mesenchymal state. In addition to these data, this study also suggests that distinct ECM rearrangements might be critical to induce p63 expression during the collective invasion process, highlighting the importance of ECM-tumor crosstalk, during breast tumor progression. Although not fully explored, it is reasonable that ECM remodeling, cellular stroma or specific signals of the TME could sustain ΔNp63 expression and activity, during the invasive process. For instance, breast cancer cells expressing ΔNp63 are reliant upon the presence of stromal cells or specific compositions of the ECM, to initiate ECM remodeling that permits a collective invasion [74,91]. In TNBC and in osteosarcoma cells, a TGFβ-rich environment modulates the pro-metastatic function of ΔNp63 [81,97]. In MCF-7 cells, an estrogen-receptor-positive breast cancer cell line, estrogen enhances cell viability and motility by inducing the ΔNp63-ITGB4 pathway [98]. In diverse basal/triple-negative breast cancer cells p63 is able to regulate the expression of milk fat globule-EGF8 (MFG-E8), which functions in this context as a pro-survival and pro-tumorigenic factor. Conversely, in ER and erbB2-positive breast cancer cells, MFG-E8 exhibits tumor suppressive functions and is not regulated by p63 [99]. Collectively, these data indicate that TME could be determinant in modulating the pro-metastatic activity of ΔNp63. On the other hand, ΔNp63 is also able to model TME, in order to create a favorable niche to sustain the metastatic capabilities of breast cancer cells. In TNBC cells, ΔNp63 directly regulates the transcription of two chemokines, CXCL2 and CCL22, which drives the recruitment of myeloid-derived immunosuppressor cells (MDSCs) [100]. MDSCs secrete pro-metastatic factors, such as MMP9 and chitinase 3-like 1, which in turn promote TNBC tumor progression and metastasis.

All together, these data suggest that ΔNp63 exploits multiple pathways, including the induction of EMT-related factors, metallopeptidases, and activation of basal epithelial program, each likely contributing to increase the invasive capabilities of breast tumors cells. Of course, genetic and molecular features or distinct TME might critically impact and influence the biological activity of ΔNp63, potentially explaining the conflicting evidence on the role that ΔNp63 plays in regulating cell motility and invasiveness in breast cancer cells.

### 4.2. ΔNp63 and Stemness of Breast Cancer Cells

As discussed in the previous paragraphs, ΔNp63 is required to maintain the self-renewing capacity of stem cells, in diverse epithelial structures, including mammary glands [101]. Based on its role in regulating mammary stem cells homeostasis, it is not surprising that the deregulated activity of ΔNp63 has been implicated in controlling the stemness properties of the breast cancer cells. By exploiting a mouse model of basal-type breast cancer tumorigenesis, Chakrabarti and colleagues identified a ΔNp63-driven pathway, which is capable of governing the tumor-initiating activity of breast cancer cells [50]. ΔNp63 depletion significantly reduces tumorsphere formation, in vitro, and decreases tumor volume in vivo. As discussed before, ΔNp63 enhances the WNT signaling, a critical regulator of epithelial stem cell homeostasis, by directly driving the expression of FZD7, a receptor for the WNT ligands. Importantly, FZD7 or ΔNp63 depletion, exerts a similar effect on tumorpheres formation, suggesting that activation of the WNT signaling is a critical effector of the ΔNp63-dependent control of breast cancer stemness, at least in basal-type breast cancer.

The prominent role of ΔNp63 in controlling the self-renewal potential and expansion of mammary cancer stem cells, has also been confirmed in a mouse model of luminal type breast carcinogenesis. Downregulation of p63 in MMTV-ErbB2-derived mammospheres, which almost exclusively express the ΔNp63 isoform, significantly limits the self-renewal capacity of cancer stem cells in vitro, and delays tumor growth, in vivo [102]. At the molecular level, ΔNp63 enhances the Hedgehog signaling, a relevant pathway in stemness regulation, by directly controlling the expression of Sonic Hedgehog (*SHH*), GLI family zinc finger 2 (*GLI2*), and Patched1 (*PTCH1*) genes.

In addition to the WNT and Hh pathway, other transcriptional targets of ΔNp63 involved in stemness regulation of breast cancer has been identified and characterized. One of these targets is the Notch1 receptor. As discussed above, in mammary glands, NOCTH signaling and ΔNp63 activity are functionally interconnected in a finely regulated cross-talk, ensuring a proper balance between the luminal commitment and maintenance of the basal cell fates. In the MCF7 breast carcinoma cell line, overexpression of ΔNp63 enhances the cancer stem cell-like features and leads to an increase of cancer cell proliferation, clonogenicity, and incidence of xenograft tumor growth, in vivo [103]. These effects have been associated with a transcriptional upregulation of NOTCH1 by ΔNp63.

Another ΔNp63 transcriptional target involved in breast cancer stemness is BMP7, a member of the bone morphogenetic proteins of the TGFβ superfamily of cytokines [104]. By using an in vitro cell system and a mouse model of breast cancer, Balboni and colleagues identified BMP7 as a bona fide ΔNp63 target gene. Notably, activation of BMP7 signaling is a common event observed in human breast cancers, mainly in the basal molecular subtype, where it can regulate EMT, epithelial cell plasticity, and tumorigenicity.

One well-established biological outcome of ΔNp63 activity in mammary gland is the modulation of ECM-mediated signaling. In normal mammary epithelial cells and in several epithelial tumors, p63 directly regulates the expression of integrin receptors (e.g., ITGB1, ITGB4, ITGA6, ITGA3), growth factor receptors (e.g., EGF-R, FGFR2), ECM components (e.g., laminin, collagen), and adhesion molecules (e.g., PERP), which ultimately might impact cancer stem cell homeostasis [68,101,105,106,107,108]. For instance, in basal-type breast tumors, ΔNp63 regulates the expression of the hyaluronic acid (HA) synthase gene *HAS3* and the HA receptor *CD44* sustaining, thus, the synthesis and the signaling of HA [109]. Inhibition of HA synthesis or, at higher extent, depletion of CD44, decreases the stem-like properties of basal-type breast cancer cells. The relevance of the HA receptor CD44 in regulating breast cancer stemness has also been confirmed by Di Franco and colleagues, which showed that in breast cancer stem cells derived from human primary tumors, ΔNp63 exerts a pro-metastatic action and enhances the stem-like features via upregulation of the *CD44v6* isoform expression [110]. These data suggest that HA metabolism and signaling is an important effector of the ΔNp63-mediated stemness control

In addition to sustain HA-mediated signaling, ΔNp63 might also favor the activation of several tyrosine kinase receptors, such as the EGF-R, whose signaling is important to sustain the proliferation and survival of cancer stem cells [111,112].

Collectively, these results clearly indicate that the pro-tumorigenic action of ΔNp63 in breast cancer is intimately linked to its ability to orchestrate several pathways, such as ECM remodeling, cytokine- or growth factors-mediated signaling, each of them contributing to maintain the stem-like features of breast cancer cells. 

## 5. Role of TAp63 in Breast Carcinogenesis

In contrast to ΔNp63, several lines of evidence have indicated that TAp63 activity might exert a tumor suppressor function in different human neoplasia, including breast carcinoma (see Figure 4). The initial observation that TAp63 might behave as tumor suppressor, emerged from the gain of function studies, which demonstrated that, in different cancer cell lines, TAp63 overexpression induces cell cycle arrest and cell death [113]. Although rare mutations of the *TP63* gene have been reported in human tumors, multiple studies have reported undetectable or very low expression of this isoform, in different human neoplasia, including invasive and metastatic lesions of mammary carcinoma [114]. Interestingly, the rare *TP63* mutations reported in squamous cell carcinoma are located in the TA domain, suggesting that inhibition of TAp63 isoform expression might favor tumor development [115]. The most compelling evidence impinging for a tumor suppressor role of TAp63, arises from the analysis of mice with genetically deleted TAp63 isoforms. TAp63 KO and, to a higher extent, TAp63 heterozygoteus mice, develop metastatic mammary and lung adenocarcinoma [116]. The anti-metastatic action of TAp63 has been further corroborated by the findings that p53 GOF mutations enhance the metastatic capabilities of tumor cells by, at least in part, inhibiting the TAp63 activity. Adorno and colleagues reported that in TNBC cells, TGFβ-mediated cell invasion is mediated by the formation of ternary complex (SMAD2-mutant p53-p63), which results in the inhibition of the transactivating action of p63, toward two p63-specific target genes, *BHLHE41* (which encodes SHARP1) and *CCNG2* (cyclin G2) [117]. Importantly, TNBC patients with a low expression of BHLHE41 and CCNG2, show a reduced survival and are at a higher risk of metastasis. The anti-metastatic action of TAp63 has been also confirmed in another study, which demonstrated that in H1299 cells, which exclusively express the TAp63 isoform, p53 mutants directly interact with TAp63 in a TGFβ-independent manner, resulting in the inhibition of integrin-α5β1 and EGF-R endocytosis cycling [118]. A further validation of the tumor suppressor function of TAp63 in breast carcinogenesis, has emerged by the finding that the in TNBC cells, the prolyl isomerase Pin1 promotes mutant p53-dependent inhibition of p63, favoring the migration and invasion of tumor cells [119]. 

In addition to SHARP1 and cyclin G2, another critical effector of the anti-metastatic activity of TAp63 is DICER, an endoribonuclease able to generate mature miRs, by processing the pre-miRs. TAp63 KO mice displaying reduced levels of DICER and murine TAp63 is able to bind to and transactivate the *Dicer* promoter [116]. The relevance of TAp63-DICER pathway has been revealed by the inhibitory effect of mutant p53 on the TAp63-dependent induction of DICER expression, and as a consequence, the reduction of the expression of certain pro-metastatic miRs, such as miR-130b and miR-200 [120].

Collectively these results indicate that during breast tumor initiation and progression, the decreased expression of TAp63 or its functional inhibition by mutant p53, might alter the expression of several TAp63 anti-metastatic target genes, thus increasing the metastatic potential of cancer cells.

As discussed before, TAp63 activity has been implicated in restraining the self-renewing ability of breast cancer cells [56]. In TAp63^−/−^ mammary adenocarcinomas or in human xenograft mammary tumors depleted of TAp63, TAp63 deficiency significantly unleashes the stemness potential of human mammary cancer cells. Although these studies unveil a potential functional link between the TAp63 tumor suppressor activity and stem cells homeostasis in breast tumors, they hardly align with previous studies showing that ΔNp63 is the only p63 isoform expressed in mammary stem cells, capable of regulating mammary stem cells activity and promoting breast cancer initiation in basal-like or luminal-like breast cancers. Likely, the analysis of tumor incidence and breast cancer stem cell activity in a mouse model of breast carcinogenesis selectively deficient for TAp63, could help clarify these controversial results.

## 6. Future Directions

TNBC is a highly malignant cancer characterized by high molecular heterogeneity and poor response to conventional therapeutic options. These clinical and molecular features negatively affect the survival rate of TNBC patients, urgently prompting to identify novel clinically actionable oncogenic routes. The transcription factor p63 has emerged as an important regulator of two highly interconnected oncogenic pathways in breast cancer, tumor cell dissemination, and stemness. The role of the *TP63* gene in breast cancer has been historically complicated by the fact that its gene codifies for two main isoforms, TAp63 and ΔNp63, having opposite functions. Although its involvement in breast cancer progression need to be further validated in mouse models of breast carcinogenesis, it seems that TAp63 might counteract ΔNp63 in controlling cancer cell stemness and tumor invasion, implying that the expression of these two isoforms need to be finely regulated during breast cancer progression. In this context, future studies should be directed to investigate the molecular pathways controlling the expression of p63 isoforms, as well as their epigenetic and transcriptome landscape in breast cancer subtypes. For instance, our knowledge about the role of p63 isoforms in modulating the expression of non-coding RNAs, is very limited. Furthermore, since TAp63α isoform needs post-transcriptional modifications to be transcriptionally active, it would be interesting to decipher the intracellular pathways controlling its anti-metastatic activity, as well as the role of other transcriptionally active TAp63 isoforms, such as TAp63γ, during breast cancer progression. On the same line, ΔNp63 expression and activity needs to be tightly regulated during breast cancer progression, especially during the collective invasion process. Although some data have suggested the importance of ECM-ΔNp63 crosstalk in tumor progression, our knowledge on the molecular pathways regulating this circuit, as well as the ΔNp63-dependent transcriptome and epigenetic landscape during collective invasion, is quite limited and represents a challenging question for future studies. Another fascinating aspect of the functional relationship between ΔNp63 and TME, regard its potential involvement in modulating the immunolandscape of TNBC and as a consequence, the potential clinical use of immunotherapy. Indeed, immunotherapy might be a promising treatment modality in TNBC, mainly in basal-like subtypes, due to their high levels of neo-antigens [15,121]. Although several evidence have indicated that ΔNp63 might transcriptionally regulate some cytokines and interleukins, the impact of ΔNp63 on modelling the immunolandscape of TNBC still needs to be elucidated. In conclusion, we believe that further investigations on the role of p63 isoforms and their connection with TME remodeling would expand our knowledge on the pathogenesis of breast tumors, potentially unveiling novel therapeutically actionable pathways.
